# Temporal Coding of Voice Pitch Contours in Mandarin Tones

**DOI:** 10.3389/fncir.2018.00055

**Published:** 2018-07-24

**Authors:** Fei Peng, Hamish Innes-Brown, Colette M. McKay, James B. Fallon, Yi Zhou, Xing Wang, Ning Hu, Wensheng Hou

**Affiliations:** ^1^Key Laboratory of Biorheological Science and Technology of Ministry of Education, Bioengineering College of Chongqing University, Chongqing, China; ^2^Collaborative Innovation Center for Brain Science, Chongqing University, Chongqing, China; ^3^Bionics Institute, East Melbourne, VIC, Australia; ^4^Department of Medical Bionics Department, University of Melbourne, Melbourne, VIC, Australia; ^5^Department of Otolaryngology, University of Melbourne, Melbourne, VIC, Australia; ^6^Chongqing Key Laboratory of Neurobiology, Department of Neurobiology, Third Military Medical University, Chongqing, China; ^7^Chongqing Medical Electronics Engineering Technology Research Center, Chongqing University, Chongqing, China

**Keywords:** temporal coding, time-variant, voice pitch contours, natural speech, inferior colliculus, fundamental frequency

## Abstract

Accurate perception of time-variant pitch is important for speech recognition, particularly for tonal languages with different lexical tones such as Mandarin, in which different tones convey different semantic information. Previous studies reported that the auditory nerve and cochlear nucleus can encode different pitches through phase-locked neural activities. However, little is known about how the inferior colliculus (IC) encodes the time-variant periodicity pitch of natural speech. In this study, the Mandarin syllable /ba/ pronounced with four lexical tones (flat, rising, falling then rising and falling) were used as stimuli. Local field potentials (LFPs) and single neuron activity were simultaneously recorded from 90 sites within contralateral IC of six urethane-anesthetized and decerebrate guinea pigs in response to the four stimuli. Analysis of the temporal information of LFPs showed that 93% of the LFPs exhibited robust encoding of periodicity pitch. Pitch strength of LFPs derived from the autocorrelogram was significantly (*p* < 0.001) stronger for rising tones than flat and falling tones. Pitch strength are also significantly increased (*p* < 0.05) with the characteristic frequency (CF). On the other hand, only 47% (42 or 90) of single neuron activities were significantly synchronized to the fundamental frequency of the stimulus suggesting that the temporal spiking pattern of single IC neuron could encode the time variant periodicity pitch of speech robustly. The difference between the number of LFPs and single neurons that encode the time-variant F0 voice pitch supports the notion of a transition at the level of IC from direct temporal coding in the spike trains of individual neurons to other form of neural representation.

## Introduction

Voice pitch is a perceptual quality of speech that varies with the fundamental frequency (F0), and is controlled by the rate of vibrations produced by the vocal folds. Natural sounds such as animal vocalizations and human speech are complex, quasi-periodic signals that can be partly characterized by their F0 and its harmonics. Periodicity in speech signals is related to the perception of low pitches (‘periodicity pitch’) (Plomp, 1967; Small, 1970; De Boer, 1976; Evans, 1978; Nordmark, 1978; Moore, 2012; Warren, 2013). It is widely known that pitch conveys essential semantic information in speech and, in tonal languages, changes in voice pitch over time can affect the meaning of words. Mandarin is a popular tonal language with four tones: flat tone, rising tone, falling then rising tone, and falling tone. For example, one syllable /ma/ pronounced with four tones: ‘mā’, ‘má’, ‘mǎ’ and ‘mà’ has four different meanings: ‘mother,’ ‘fiber,’ ‘horse’ and ‘scold’, respectively.

Previous studies have suggested that the phase-locked neural temporal activity of auditory nerve (AN) fibers and cochlear nucleus (CN) neurons plays a crucial role in representing periodicity pitch. The neural responses in these structures support the *predominant interval hypothesis* of pitch coding, i.e., the most frequent interspike interval exhibited by large samples of neurons (population interval distributions) corresponds to the pitch heard (Licklider, 1951; van Noorden, 1982; Rhode, 1995; Cariani and Delgutte, 1996a,b; Sayles and Winter, 2008; Moore, 2012). The inferior colliculus (IC) in the brainstem plays a key role in the auditory pathway, and receives input from several peripheral auditory nuclei as well as projections from auditory cortex. While most neurons in AN and CN can phase lock to stimulus periodicity, only 68% of IC neurons displayed phase-locked activity in response to pure tones (Liu et al., 2006). Responses to pure tones and amplitude modulated signals display a similar central decrease the upper frequency limit of phase-locking from the AN, through the IC, and finally to the auditory cortex. For example, the upper frequency limit for phase-locking to pure tones in guinea pigs decreases from 3.5 kHz in the AN (Palmer and Russell, 1986), to 2 kHz in the ventral CN (Winter and Palmer, 1990), to 1.5 kHz in the dorsal CN (Goldberg and Brownell, 1973), to 1 kHz in the IC (Liu et al., 2006), to 1 kHz in the medial geniculate body (Wallace et al., 2007), and to 250 Hz in primary auditory cortex (A1) (Wallace et al., 2002). It has also been reported that the spike rate of IC neurons tunes to the modulation rate of amplitude modulated signals (Langner and Schreiner, 1988; Krishna and Semple, 2000), suggesting the existence of a periodicity modulation map in the IC (Schreiner and Langner, 1988; Langner et al., 2002; Schnupp et al., 2015). Langner and Schreiner (1988) examined barbiturate-anesthetized cat IC neurons’ responses to amplitude modulated signals, and demonstrated that the upper limit of modulation frequency synchronization was 600 Hz. These authors also found that the number of single neurons tuned to a given best modulation frequency was 75% (tested by firing rate) and ∼33% (measured by synchronization index). Together, these findings suggest that neural response patterns capable of supporting both rate and temporal coding of periodicity exist at the level of the IC. The upper limit of modulation frequency synchronization also declines from auditory periphery to auditory cortex (Palmer, 1982; Schreiner and Urbas, 1988; Rees and Palmer, 1989; Joris and Yin, 1992, 1998; Rhode and Greenberg, 1994; Bieser and Müller-Preuss, 1996; Eggermont, 1998; Joris et al., 2004), and neurons represent modulation frequency using rate and/or sparse timing representation in the auditory cortex (Lu et al., 2001; Wang et al., 2003; Lu and Wang, 2004; Bendor and Wang, 2007; Malone et al., 2007; Bendor and Wang, 2008). Studies have also shown that neural mechanisms coding periodicity have a temporal-to-rate coding transformation in thalamus and cortex (Bartlett and Wang, 2007; Wang et al., 2008; Bendor and Wang, 2010). The spike rate of pitch-selective neurons can also represent the periodicity pitch of temporally regular sounds in the low frequency region of auditory cortex in marmosets (Bendor and Wang, 2005, 2010). Together, these studies suggest that the temporal representation of periodicity pitch may be transformed to a rate-based representation (Bartlett and Wang, 2007; Wang et al., 2008; Bendor and Wang, 2010; Plack et al., 2014; Langner, 2015) or spatial-temporal representation (Loeb et al., 1983; Oxenham et al., 2004; Bernstein and Oxenham, 2005) in the upper auditory pathway.

Scalp-recorded frequency following responses (FFRs) reflect sustained phase-locked activity of populations of neurons (Worden and Marsh, 1968), and are generated predominately from the IC (Smith et al., 1975; Chandrasekaran and Kraus, 2010). FFRs have been proposed to represent periodicity pitch of speech (Greenberg, 1981). For example, Krishnan et al. (2004) recorded FFRs to Mandarin speech sounds and found that the phase-locked interpeak intervals of the FFR waveform robustly matched the time-variant fundamental period of the stimuli. Gockel et al. (2011) also found that scalp-recorded FFR responses in humans were similar to modeled neural responses in the auditory periphery, and did not necessarily reflect any additional pitch-related processing. However, the periodicity strength of FFR was stronger for people with tonal language than English (Krishnan et al., 2005, 2010; Bidelman et al., 2011; Chandrasekaran et al., 2012), for people who are musicians compared to no musical experience (Wong et al., 2007; Bones et al., 2014), for adults compared to neonates (Jeng et al., 2011), and after musical training compared to before (Song et al., 2008). Because it is hard to control for these factors, human studies are limited in their ability to explore neural coding mechanisms, while studies using animal models can provide more detailed and comprehensive information to evaluate the neural mechanisms of coding pitch information. In the present study, neuronal activity in guinea pig IC was measured to investigate how it represent the time varying periodicity pitch of natural speech. To our knowledge, this is the first study to explore the correspondence between the temporal information of the single IC neurons with the time varying F0 contours in Mandarin speech.

Neural activity measured from an electrode in the IC includes two components: local field potentials (LFPs), which are dominated by the synaptic input information, and action potentials from single neurons or multiple neurons, which reveal axonal output information (Pettersen et al., 2012). LFPs can be recorded in IC with high spatial resolution and reflect neural ensembles with synchronized synaptic inputs in a small area around the electrode (Kraus et al., 1994; King et al., 1999). LFPs recorded in guinea pig IC have shown phase-locking to the acoustic characteristics of steady state vowels and formant transition periods of synthetic speech sounds such as the vowel-consonant-vowel token /ada/ (Cunningham et al., 2002). White-Schwoch et al. (2016) recently compared the waveforms of multi-unit activity recorded in the central nucleus of guinea pig IC and scalp-recorded FFRs in humans in response to same speech sound /da/. The authors showed that the responses’ waveforms were morphologically similar and contained a periodic component corresponding to the periodicity of stimuli. In the light of these studies, we hypothesized that the LFPs of the IC could represent the time-variant periodicity pitch of speech.

Compared with LFPs recorded in the IC which are dominated by the input to the IC, spiking activity recorded from the IC reflects the output information of neurons processing pitch information (Pettersen et al., 2012). Studies have shown that the discharge pattern of IC neurons synchronize to the F0 of harmonic tones. For example, Sinex and Li (2007) measured IC single neuron responses to a single harmonic tone, and found that the response envelope of some neurons modulated at an F0 of 250 Hz. Shackleton et al. (2009) also found that the temporal patterns of most IC multi-neuron clusters significantly synchronized at a frequency equal to F0 (at and above 282.8 Hz).

In contrast to synthesized harmonic tones with a fixed F0, animal vocalizations and human speech are amplitude and frequency modulated. For example, guinea pigs communicate using a characteristic ‘whistle’ – a vocalization with patterns of amplitude and frequency modulation (FM). Studies have demonstrated that neural responses in the IC can reflect the direction of amplitude and FMs (Suta et al., 2003; Woolley and Casseday, 2005). For instance, Suta et al. (2003) found that single IC neurons in guinea pig had a significantly stronger response to natural whistles compared to time-reversed whistles. Some neurons in the IC of bat are highly sensitive to the direction of FM (Brimijoin and O’Neill, 2005; Andoni et al., 2007; Williams and Fuzessery, 2010).

Studies have confirmed that spike activity patterns of IC neurons in animals are sufficiently abundant to explore the processing of speech (Perez et al., 2013; Steadman, 2015). For instance, Ranasinghe et al. (2013) found that IC neuronal activity patterns became more diverse when the speech added period information, and suggested that activity was determined by the F0 of speech.

In summary, previous research suggests that spike-timing information of single IC neurons could represent the periodicity of stimuli with a fixed F0, and the periodicity coding of IC neurons are affected by the stimulus type (i.e., click trains, sinusoidal amplitude modulated noise and iterated rippled noise) (Schnupp et al., 2015). However, how dynamic F0 patterns (such as are present in tonal languages) are encoded by IC neurons is currently unknown.

The aim of this study was to determine how the IC encodes time-variant F0 contours in Mandarin tones. There were two objectives for this study. The first objective was to investigate how the phase-locked activity underlying LFPs could represent the periodicity pitch and spectral components of natural speech. The second objective was to examine whether spike-timing information from single IC neurons could dynamically phase-lock to the time-variant F0 contours of natural speech. To address these aims, LFPs and single neuron activity were simultaneously recorded in the IC of anesthetized guinea pigs.

## Materials and Methods

Six healthy adult guinea pigs of either sex (weight 250–450 g) were used in this study. All guinea pigs were purchased from the Experimental Animal Centre of Chongqing Medical University. The procedures were performed in accordance with protocols of the Care and Use of Laboratory Animals approved by the Third Military Medical University.

### Animal Anesthesia and Surgery

Surgical and neural data collection methods have been described previously (Peng et al., 2016). Healthy animals were maintained at a surgical level of anesthesia by an initial intraperitoneal injection of urethane (1.2 g/kg body weight in 20% sterile saline). Supplemental injections of urethane (0.16 g/kg) were administrated on indication of a paw withdraw reflex every 30–60 min. The body temperature of animals was maintained at 38°C by using a thermostatic bath (HSS-1B, Chengdu Instrument Factory, Chengdu, China).

The animal was placed inside a sound-attenuating room, which the ambient noise floor was 25 dB sound pressure level (SPL). Before surgery, auditory brainstem responses (ABRs) were recorded to assess the hearing sensitivity of animals. ABRs were evoked by a click stimulus (100 μs rectangular pulse) at a repetition rate of 4 Hz, and 600 clicks were presented at every intensity level (30 dB SPL to 80 dB SPL in 10 dB steps) in each animal. Stainless steel needle electrodes positioned at the vertex in the middle of ears, and the mastoid of the ipsilateral ear and nose served as active, reference and ground electrodes respectively. Normal hearing sensitivity was confirmed if the threshold of ABR was at or below 30 dB SPL.

After confirmation of normal hearing, a midline incision was made along the scalp surface, and the dorsal surface of the skull was exposed. The head was fixed in place anterior to the bregma by a custom head holder, which was held at its bottom by using 3 small screws and attached by dental acrylic. The right temporalis muscle was retracted and removed, and an approximate 5 × 5 mm opening was drilled in the skull dorsal to the temporoparietal suture and rostral to the tentorium (Snyder et al., 2004). The dura mater was then removed and the cortex was reflected and aspirated, allowing direct visualization of the surface of IC.

### Acoustic Stimulation

**FIGURE 1:**
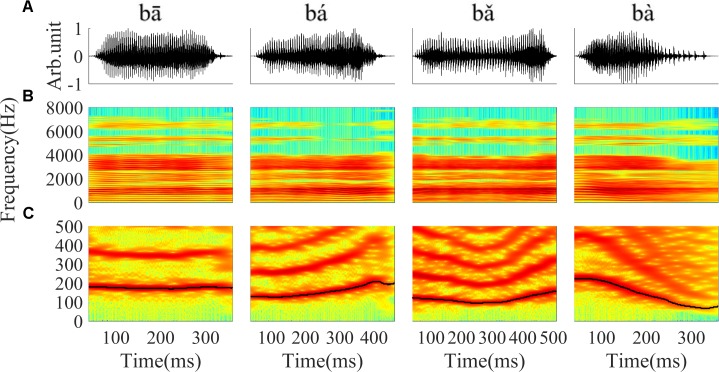
The waveform, spectrogram and F0 curve of the four Mandarin tones used in the current study. **(A)** From left to right, waveform of ‘bā’,‘bá’, ‘bǎ’, ‘bà’ stimulus, respectively. **(B)** Spectrogram of ‘bā’, ‘bá’, ‘bǎ’, ‘bà’. **(C)** The low frequency (0–500 Hz) spectrogram with the F0 curve shown by a black line. The colors in the spectrogram represent spectral energy from blue (low) to red (high).

Stimuli were four Mandarin words downloaded from the database of standard Mandarin speech (Mandarin, 2015) and the sample rate was 16 kHz. The four words were one syllable /ba/ pronounced by a male speaker with four lexical tones: bā ‘eight,’ bá ‘pluck,’ bǎ ‘target,’ bà ‘father.’ The waveform and spectrogram of the speech used in this study are displayed in **Figure 1**; the duration of stimuli ranged from 400 to 600 ms. The F0 contours (**Figure 1C**) were extracted from the spectrogram by calculating the peak spectral energy in a sliding window (80 ms Hanning window shifted in 1 ms step). The F0 of the four tones varied with time (**Figure 1C**). The flat tone had a steady F0 of 170 Hz, the rising tone F0 increased from 120 to 210 Hz, the falling then rising tone decreased from 120 to 90 then increased to 160 Hz, and the falling tone decreased from 220 to 80 Hz.

Sound stimuli were presented by a laptop using LabVIEW, converted by a 16-bit D/A converter (DAQ 9264; National Instruments) and delivered in free-field conditions from an earphone equipped with a custom-made box (Beyerdynamic DT 770, Germany). The earphone was located 1cm away from the animal’s ear canal contralateral to the exposed IC. The acoustic delivery system was calibrated (Tao et al., 2016) using a ¼ microphone and amplifier (Piezotronics Inc., United States) to deliver a flat response (±50 dB) across the frequencies (0.5–8 kHz) used in this study.

Tone bursts (duration 12 ms with 2 ms rise/fall ramps, frequency from 0.5 to 8 kHz steps 1/8 octave) were presented to estimate the characteristic frequency (CF) of the single neuron and LFP. Twelve levels were presented at each frequency (from 30 to 80 dB SPL in 5 dB steps). For each penetration into IC, neuronal responses to speech were obtained from 100 to 1000 ms relative to stimulus onset. All speech stimuli were presented at a level of 80 dB SPL, and delivered in pseudorandomized order once every 2 s. Each stimulus was presented either 9 (for 39 out of 90 recording sites) or 10 times.

### Neuronal Activity Recording

Extracellular neuron activity and LFPs were simultaneously collected using a single channel tungsten electrode (1–3 MΩ, Global Biotech INC., China), and the ground electrode was placed at the temporal bone below the skin. The electrode was mounted and controlled by a microdrive (50-12-1C Hydraulic Probe Drive and 50-12-9 Manual Drum Unit, FHC, United States), and advanced in a dorso-ventral direction with 1 μm steps. Bursts of broadband white noise (duration 50 ms, 10 ms rise/fall ramps) presented at 4 Hz were used as the search stimulus and single neurons were identified by visual inspection of the response waveform morphology (Palmer et al., 2013).

Several parallel tracks were made in sagittal or coronal planes of the IC for each animal. Single neuron activity was amplified by a headstage connected with a Cerebus multichannel physiological signal acquisition system (Cerebus 6.01, Blackrock Microsystems, Salt Lake City, UT, United States). Single neuron spikes were band-pass filtered between 250 and 5 kHz and sampled at a rate of 30 kHz. For LFPs, the signal was band-pass filtered from 50 to 500 Hz and at a sampling rate of 2 kHz.

### Data Analysis

All subsequent data analyses were processed offline in Matlab 2016b.

#### LFPs of IC Recordings

In order to verify whether the recorded LFPs in response to speech originated from IC, the latency between each LFP and a low pass filter version of a given stimulus was examined. The low-pass filter was an eighth order digital Butterworth filter with cut-off frequency at 500 Hz and was applied in both forward and reverse directions to minimize phase distortions. The latency of each LFP was defined as the time lag corresponding to the peak of the cross-correlation (LFPs and low pass filtered version of stimuli) function.

The biphasic waveform of LFPs in response to a pure tone have an upward-going shape with a first positive peak at ∼6.5 ms and duration of ∼30 ms (Orton et al., 2012). For each LFP, the response area was constructed by calculating the peak-to-peak amplitude in the response duration after the stimulus onset for each frequency and stimulus level combination (Fallon et al., 2016). The CF was defined as the frequency which evoked a detectable peak above the background noise at the lowest stimulus level.

To visualize the time-varying periodicity of LFPs corresponding to the F0 of each stimulus, an autocorrelogram was constructed using a periodicity detection short-term autocorrelation algorithm (Krishnan et al., 2004). The algorithm calculated the cross-correlation function within a rectangular window (40 ms window duration shifted in 1 ms steps) between the signal (LFPs and low pass filtered stimuli) and the copy signal shifted in 0.2 ms steps. In the resulting autocorrelogram, the vertical axis shows the time lag between the original signal and its copy signal, and the horizontal axis shows the response time relative to the onset. Colors represent the degree of correlation function between the signal and its copy at each response time and corresponding time lag.

To quantify the degree of periodicity in the neural activity underlying the LFPs, the pitch strength was calculated. The pitch strength at each time window (40 ms) was the maximum correlation magnitude (LFPs and copy LFPs) at the time lag corresponding to the stimulus F0 (converted to a period) in the same time window. The overall pitch strength of each LFP was calculated by averaging the pitch strength measures over all time windows. A one way analysis of variance (ANOVA) was computed to examine whether the pitch strength measure was different for each of the four tones.

To examine whether the LFPs could represent the time-variant F0 and spectral components of a stimulus, *narrow-band spectrograms* of the stimuli and LFPs were compared. Narrow-band spectrograms were generated by calculating the short-term Fourier transform of 80 ms sliding hamming windowed LFPs and the stimulus waveforms, shifted in 1 ms steps. The windowed signal was zero-padded to 250 ms before the Fourier transform; as a result, the frequency resolution was 4 Hz.

In order to quantify the degree to which the LFPs represented the F0 dynamically, the *stimulus-to-response correlation* was calculated (Pearson’s correlation coefficient *r*). We calculated the cross-correlation between the F0 contours extracted from the narrow-band spectrogram of each response and its corresponding stimulus. This measure provides both the strength and direction of the relationship between two signals. The F0 curve was extracted from the spectrogram of the LFP responses by finding the maximum spectral magnitude in predefined frequency ranges (F0 ± 50 Hz).

To further quantify the accuracy of LFPs coding F0 across stimulus duration, the *linear pitch tracking error* was computed by finding the absolute difference between stimulus F0 and response F0 at each corresponding time bin then averaging them across all time bins (Song et al., 2008).

#### Single Neuron Activity

For each single neuron, a frequency response area (FRA) was created with a procedure that delivered tone bursts at different frequencies and intensities. The FRA of each single neuron was calculated as the driven spike rate (the recorded spike rate minus the spontaneous spike rate) in a 50 ms window following tone burst onset. Spontaneous spike rate was calculated in 50 ms window before the tone burst onset. CF was determined as the frequency of a tone burst that evoked a detectable response at the lowest stimulus level.

To visually evaluate whether the most frequent interspike intervals of single neuron corresponded to the fundamental period, *all-order intervals* (time domain) were calculated using the intervals between both successive and non-successive spikes. The method was used by Cariani and Delgutte (1996a) to evaluate AN fibers in response to vowels with time-variant F0s. For each spike in a single stimulus presentation, all interspike intervals preceding the reference spike were calculated and listed. The running all-order intervals thus incorporate peristimulus time and interspike interval information.

To visualize whether the post-stimulus time histogram (PSTH) of single neurons could represent the F0 contours, a spectrogram of the PSTH was plotted. Spectrograms were constructed by calculating the short-term Fourier transform for 80 ms sliding hamming windowed PSTHs shifted in 1 ms steps. The signal was zero-padded to 250 ms, and the frequency resolution was 4 Hz.

To examine whether the single IC neuron discharge pattern was synchronized to the stimulus F0, the PSTH and the *response spectrum* were computed (Miller and Sachs, 1984). The PSTH and response spectrum were generated from spikes occurring at least 50 ms after stimulus onset to exclude the onset response. For each speech stimulus, the PSTH was divided into several successive time segments (256 bins per 50 ms). To examine the response frequency components, the discrete Fourier transform of the PSTH at each time segment was calculated. To increase frequency resolution, each 50 ms time segment was zero-padded to 200 ms before calculating the discrete Fourier transform; as a result, the frequency resolution was 5 Hz. The synchronization index (SI) was used to quantify the degree to which a single neuron’s spiking pattern represented all stimulus frequencies. The SI was computed as the magnitude of the Fourier component normalized by the average firing rate across each 50 ms window (Miller and Sachs, 1983; Sinex and Li, 2007; Sinex, 2008). The SI is an equivalent measurement to vector strength (Syka and Aitkin, 1981; Nakamoto et al., 2010). The value of SI can change between 0, representing no synchronization, and 1, for perfect synchronization. The statistical significance of synchronization was tested using Rayleigh values (2*nr*^2^, where *n* is the number of spikes in the 50 ms time segment, and *r* is the SI) when Rayleigh values were >13.8 (*P* < 0.001) (Mardia, 1975).

## Results

### Local Field Potentials

Simultaneous recordings of LFPs and single neuron spike activity at 90 IC sites were carried out in six guinea pigs. The CF of LFPs ranged from 500 to 8000 Hz (**Table 1**), and they are all larger than the stimuli F0 (80–220 Hz).

**Table 1:** The distribution LFPS according to CF.

CF range (kHz)	0.5–1	1–2	2–4	4–8
No. of neurons	14	24	28	24


**FIGURE 2:**
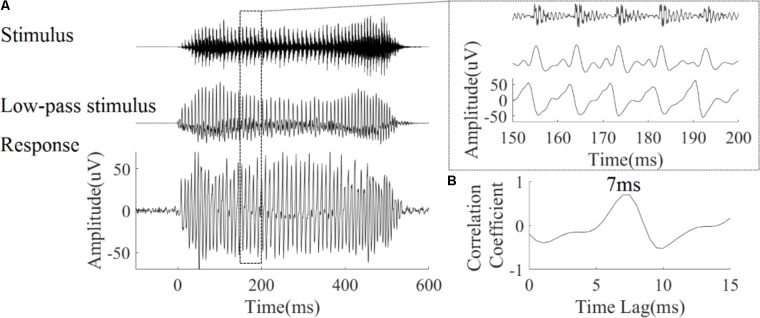
**(A)** Stimulus waveform (top), low-pass filtered stimulus waveform (middle) and one representative trace of an LFP response (CF = 5.19 kHz) waveform (bottom) to the flat tone stimulus ‘bā’. The right panel shows a magnified version from 150 to 200 ms. **(B)** The cross-correlation between the low-pass stimulus and response; the peak corresponded to a latency of 7 ms.

To test the relationship between the LFPs and the stimulus waveform, we compared the waveform of the flat tone stimulus, a low-pass filtered version of the stimulus and a typical trace of the LFPs recorded in the IC (**Figure 2A**). The LFP waveforms were morphologically similar to the low pass filtered stimulus waveform, suggesting that the LFPs were phase-locked to the stimulus (**Figure 2A** right panel in dashed rectangular).

#### Verification of LFPs Source

To verify that the LFPs originated from the IC rather than far-field sources, the latency of all recorded LFPs was calculated. The latency of each LFP corresponded to the peak of the cross-correlation function between the LFP waveforms and low-pass filtered stimulus waveforms (**Figure 2B**). On average, the LFP latency [mean = 6.6 ms, standard deviation (SD) = 1.5 ms] was within the previously described for guinea pig IC (5–6 ms) (Harrison and Palmer, 1984; Orton et al., 2012).

#### LFPs Represent Time-Variant Periodicity in the Stimulus

**FIGURE 3:**
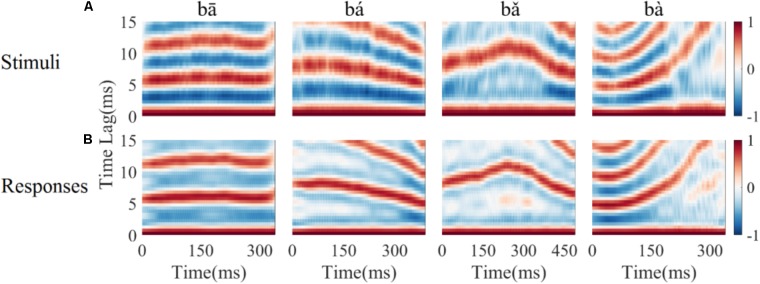
Autocorrelograms of the stimuli (row **A**), and one representative LFP response (CF = 5.19 kHz) (row **B**). The time indicated on the horizontal axis represents the start of each 40 ms time bin calculated, the vertical axis represents the time lag (ms) between original signal and a time shifted copy signal, and colors represent the strength of correlation (red is positive, blue is negative).

Autocorrelograms were used to visualize the periodicity of signal. Autocorrelograms represent the degree of correlation between the signal and its copy at each response time and corresponding time lag. As shown in **Figure 3A**, bands with high correlation values can be seen, and reflect the fundamental period of the stimulus (or its multiples). **Figure 3B** shows the same analysis applied to each of the four LFP responses. There is a high degree of correspondence between the autocorrelogram for each stimulus (**Figure 3A**) and autocorrelograms calculated from the corresponding LFP response (**Figure 3B**).

To quantify the degree to which periodicity in the LFP responses follows the fundamental period of the stimulus, the pitch strength of each LFP was computed (section 2.4.1.4). Pitch strengths for an example LFP were 0.76, 0.69, 0.68, and 0.59 for the four tones respectively (**Figure 3**). The pitch strength (mean ± SD) of all recorded LFPs for each of four tones was 0.5 ± 0.15, 0.53 ± 0.13, 0.61 ± 0.07, and 0.4 ± 0.09, respectively, and was significantly correlated with the CF (*r* = 0.56, 0.30, 0.32, 0.44, *p* < 0.05 respectively). The tone type had a significant effect on the pitch strength as determined by a one way ANOVA [*F*(3,356) = 36.46, *p* < 0.0001). A *post hoc* Tukey–Kramer test demonstrated that the pitch strengths of the rising tone and the falling then rising tone (both included a rising F0) were significantly larger than the flat tone and the falling tone (*p* < 0.001).

#### Spectrogram of LFPs Represent Time-Variant Frequency Components

**FIGURE 4:**
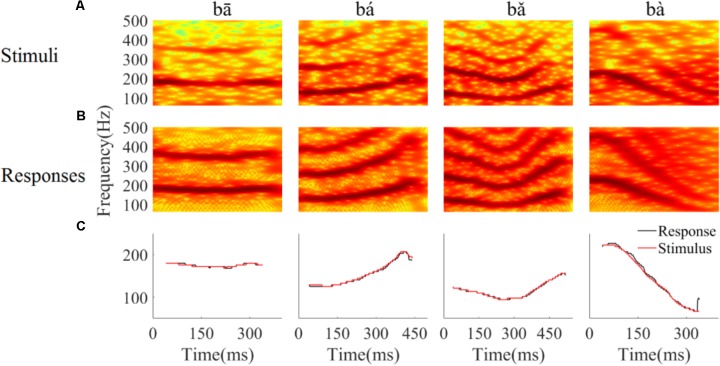
Spectrograms of the stimulus **(A)** and single representative LFP recording (CF = 2.38 kHz) **(B)**. **(C)** F0 curve of stimulus (red) and response (black). From left to right, each column corresponds to flat tone, rising tone, falling then rising tone and falling tone, respectatively. In rows **(A,B)**, the horizontal axis indicates the midpoint of each 80 ms hanning window, vertical axis indicates frequency, and the colors indicate spectral energy (red is highest). In row **(C)**, the horizontal axis represents the midpoint of each time bin, the black line represents the response F0 curve and red line represents the stimulus F0 curve.

To determine whether the LFPs represent the time-variant frequency components in the stimuli, the spectrograms of single LFP responses and the stimuli were compared (**Figure 4**). The spectrogram of single LFP responses (**Figure 4B**) show high spectral energy bands at the stimulus F0 and its harmonics (**Figure 4A**). For example, there are four time-variant spectral energy bands in the spectrogram of the falling then rising stimulus (**Figure 4A**, third column), and corresponding energy bands at the same frequencies are also evident in spectrogram of the response (**Figure 4B**, third column). When the spectrogram of all recorded LFPs was plotted (for all recording sites), some spectrograms only showed the spectral band at F0, but not at the harmonics. However, it is clear that the spectral bands in the LFPs follow the rising/falling dynamics of the changing F0 in the stimuli. The stimulus F0 (red line) and corresponding response F0 contours (black line) extracted from the spectrogram of an example LFPs are shown in **Figure 4C**. The correlation and linear pitch tracking error between the stimuli F0 contours and the corresponding response F0 contours were used to quantify the degree to which the LFP response tracked the direction and accuracy of the time-variant stimulus F0, respectively. The stimulus-to-response correlation for each tone of this example LFP was 0.86, 0.99, 0.99, and 0.99, respectively. The linear pitch tracking error of the representative LFPs for each tone was 0.96, 1.77, 1.34, and 3.4 Hz, respectively.

To quantify whether all recorded LFPs could match the time-variant F0 contours robustly, the stimulus-to-response correlation was computed individual for each LFP. The stimulus-to-response correlation (mean ± SD) for flat tone, rising tone, falling then rising tone, and falling tone stimulus was 0.66 ± 0.27, 0.93 ± 0.08, 0.95 ± 0.07, 0.98 ± 0.03 respectively, and 93% (84 of 90) of the cross-correlations between the stimulus and response F0 contours were significant (*p* < 0.05).

To assess whether all recorded LFPs could code the stimulus F0 accurately, the linear pitch tracking error was calculated. Across all recordings, the linear pitch tracking error (mean ± SD) of flat tone, rising tone, falling then rising tone and falling tone was 3.29 ± 4.33 Hz, 5.37 ± 3.25 Hz, 2.59 ± 1.79 Hz, and 6.64 ± 3.09 Hz, respectively. The maximum linear pitch tracking error was calculated for each LFP in response to the four tones. The maximum linear pitch tracking error was lower than 5 Hz in 26 out of the 90 LFPs and lower than 10 Hz in 67 out of the 90 LFPs.

### Single Neuron Activity

**FIGURE 5:**
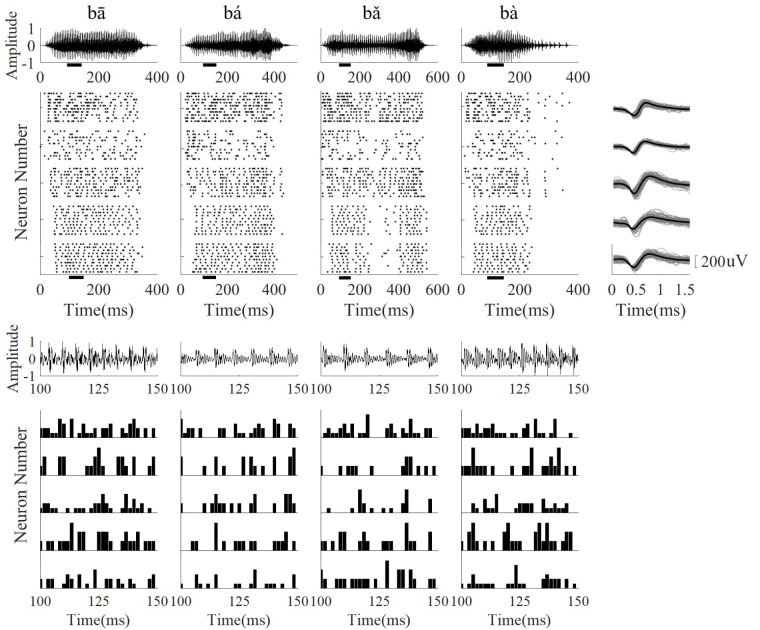
The stimulus waveform **(top row)**, five representative neuron raster plots (from **top** to **bottom**, CF was 2.59, 5.19, 6.73, 2.59, 5.19 kHz, respectively), and corresponding neuron spike wavefoms **(right column)**. The PSTH of each neuron in a short time segment (100–150 ms) is shown in the bottom panel. From **(left)** to **(right)**, the stimulus waveforms were ‘bā’,‘bá’, ‘bǎ’, ‘bà’, respectively. For the spike waveforms, the gray lines represent each single spike waveform and the black line represents the mean waveform. The short black bars below the horizontal axis in the stimulus waveform and raster plots represent the time segment used for calculating the PSTH.

Single neuron activity in response to 10 presentations of each speech stimulus was recorded. The raster plots of five neurons with a sustained response to speech and the corresponding spike waveforms are displayed in **Figure 5**. The PSTHs of each neuron at one time segment are showed in **Figure 5** (bottom panel), and the neurons only phased locked to the some of the stimulus periods. Responses were mostly stimulus-locked and the response duration was similar to the stimulus time. The CFs of single neurons was larger than the stimulus F0 (80–220 Hz) and ranged from 500 to 8000 Hz. The distribution of single neuron CFs at each frequency range was the same as the distribution of LFPs CF (**Table 1**).

#### Running All-Order Intervals (Time Domain)

**FIGURE 6:**
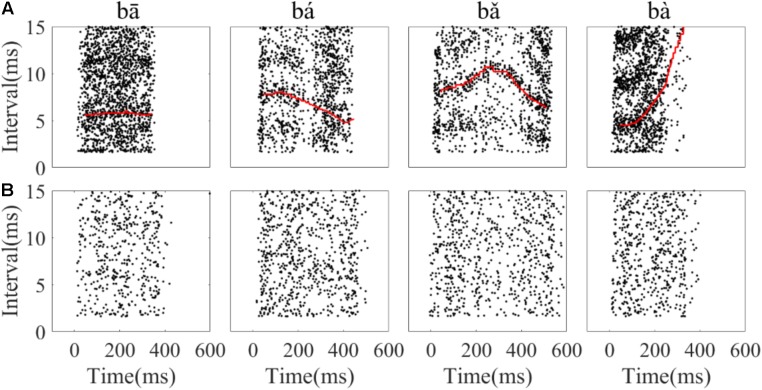
Example of running all-order intervals of two representative IC neurons’ responses to the four speech stimuli. **(A)** One neuron (CF = 2.83 kHz) in which the most frequent intervals followed the corresponding fundamental period of the stimulus (red line in each figure). **(B)** One neuron (CF = 3.08 kHz) with intervals that did not match the F0 period. From left to right, each figure corresponds to stimulus ‘bā’,‘bá’, ‘bǎ’, ‘bà’, respectively. Each dot represents an interspike interval (vertical axis) at specific end time (horizantal axis) relative to stimulus onset.

To assess whether the temporal information of single neuron spikes could represent the F0 contours of Mandarin tone stimuli, running all-order interval distributions of all neurons were constructed. Running all-order intervals from two representative neurons in response to the four speech stimuli are displayed in **Figure 6**. Bands of most frequent interspike intervals for one neuron (**Figure 6A**) that match the time-variant fundamental period of the stimuli can be seen (red line in **Figure 6A**). Particularly, a band of most frequent interspike intervals for the rising tone decreased from 8 to 5 ms (**Figure 6A**, second column), and in the falling then rising tone increased from 8 to 11 ms then decreased to 6 ms (**Figure 6A**, third column). Specifically, two most frequent interspike interval bands in the flat tone (**Figure 6A**, first column) and the falling then rising tone (**Figure 6A**, third column) are evident, and the top interspike interval band corresponds to twice fundamental period. By visual inspection of the running all-order intervals for all neurons, only five neurons showed such clear bands of most frequent interspike intervals related to the fundamental period of the stimulus. The all-order intervals for most neurons did not clearly reflect the fundamental period (an example of another neuron with a similar CF is shown in **Figure 6B**).

#### PSTH, Spectrogram of PSTH and Response Spectrum (Frequency Domain)

**FIGURE 7:**
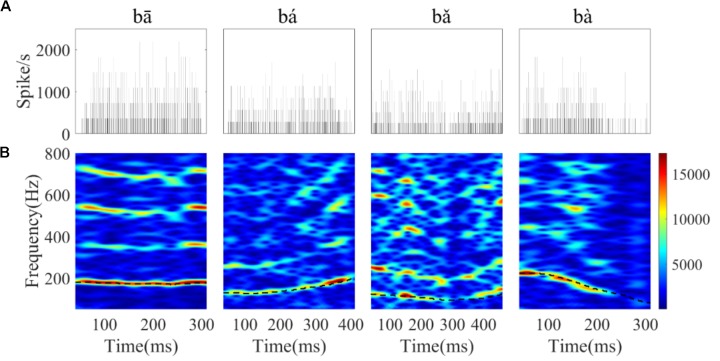
The PSTHs **(A)** and spectrograms of the corresponding PSTH **(B)** of a single neuron (CF = 8 kHz) in response to the four tones. The horizontal axis of the spectrogram indicates the midpoint of each 80 ms hanning window, the vertical axis indicates frequency, and the colors indicate the amplitude of the Fourier transform at each time bin (red is highest). The black dashed line in each figure corresponds to the stimulus F0 curve.

Narrow-band spectrograms of the PSTHs were used to visualize whether the discharge pattern of single neurons could represent the F0 contours and harmonics of the stimuli. One example of a single neuron’s PSTHs (**Figure 7A**) and its corresponding spectrograms (**Figure 7B**) are displayed. Bands of high spectral energy can be clearly seen that correspond to the F0 contour (black dashed line) of the flat tone, the rising tone and the falling tone (**Figure 7B**, first, second, and fourth column). Harmonics can also be seen for the flat tone (**Figure 7B**, first column). However, the bands of high spectral energy in the falling then rising tone were not continuous throughout the response duration of this neuron (**Figure 7B**, third column).

**FIGURE 8:**
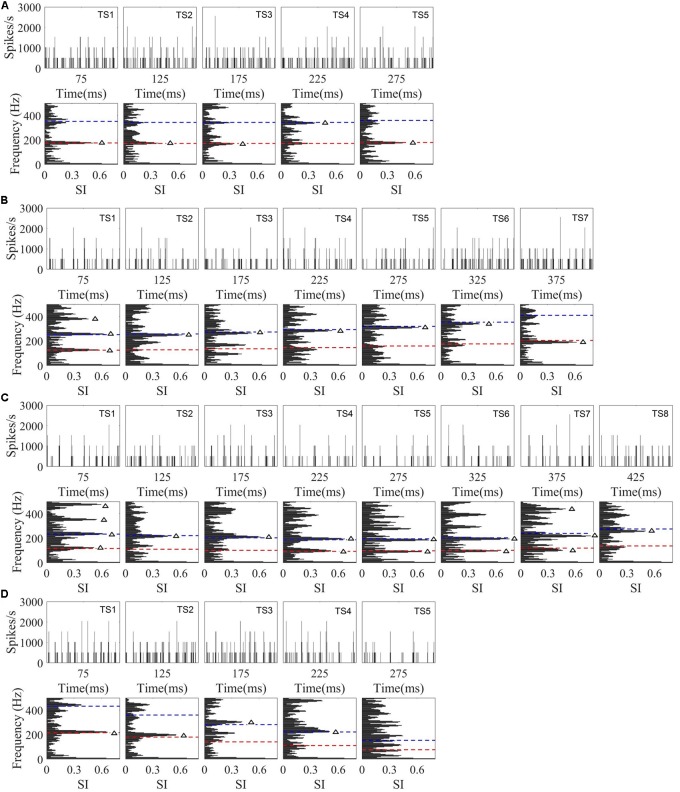
Example of the PSTHs at each time segment, and PSTH response spectra from a single neuron (CF = 5.66 kHz) response to four tones. **(A–D)** Correspond to flat tone, rising tone, falling then rising tone, and falling tone, respectively. For each PSTH, the horizontal axis represents time relative to onset of speech, the vertical axis indicates the spike rate per 0.195ms bin, and the time segment (TS) is indicated in the right corner of each plot. The stimulus F0 (red line) and 2F0 (blue line) are plotted in each response spectrum. The triangle symbol in the response spectrum represents a significantly synchronized frequency.

To examine whether the discharge pattern of each single IC neuron would significantly correlate with the time-variant stimulus spectral components, the response spectrum of successive 50 ms time segments of the single-unit’s PSTH were calculated. The PSTHs and response spectrum for the four stimuli from one representative neuron (CF = 5.66 kHz) are plotted in **Figure 8**. Frequency components at which the Raleigh test indicated that the neural spiking pattern was significantly synchronous are indicated with triangle symbols. In this single representative neuron, we found that the significantly synchronized frequency in the response spectrum corresponded to the stimulus F0 (dashed red line), 2F0 (dashed blue line), or other harmonics [**Figures 8B,C** TS (time segment) 1]. For the flat tone (**Figure 8A**), we found that the significantly synchronized frequency corresponded to F0 in four of the five time segments, and 2F0 in one of the five time segments (**Figure 8A**, TS4). However, for the rising tone (**Figure 8B**), only two of the seven time segments had significantly synchronized frequencies at F0 (**Figure 8B** TS1 and TS7), with activity in the remaining five time segments corresponding to 2F0. For the falling then rising tone (**Figure 8C**), the significantly synchronized frequencies correspond to both the F0 and 2F0 (**Figure 8C** TS1, TS4, TS5, TS6, TS7). For the falling tone (**Figure 8D**), there was no significantly synchronized frequency in the last time segment (**Figure 8D** TS5).

**FIGURE 9:**
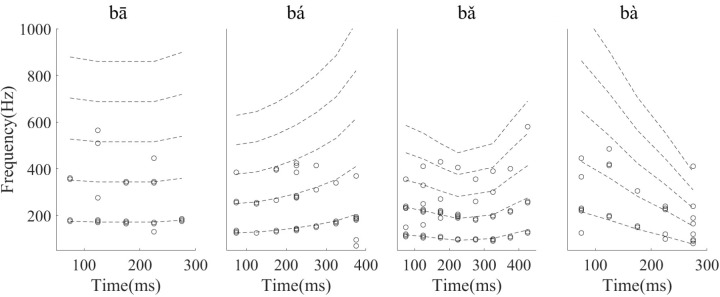
The significant synchronization frequency component with maximum synchronized index for neurons with at least one significantly synchronized frequency (*n* = 42). Each circle indicates one neuron significantly synchronized frequency at each time segment, and the dashed line indicates the stimulus F0 and harmonics. The time indicated in the *x* axis is the midpoint of each time segment.

The results above were descriptive for a single neuron. To test whether the discharge pattern of *all* recorded single neurons could significantly synchronize to the stimuli F0s or harmonics, the significantly synchronized frequency components with the largest SI (47% of neurons) were computed and are shown in **Figure 9**. The stimuli F0s and their harmonics are shown with dashed lines. Twenty-four percent of 90 neurons had a significantly synchronized frequency in more than 5 time segments for all stimuli. The significantly synchronized frequency (open circles) was mostly at F0 (67%) or 2F0 (25%), and only a small number (7%) at other harmonics.

**FIGURE 10:**
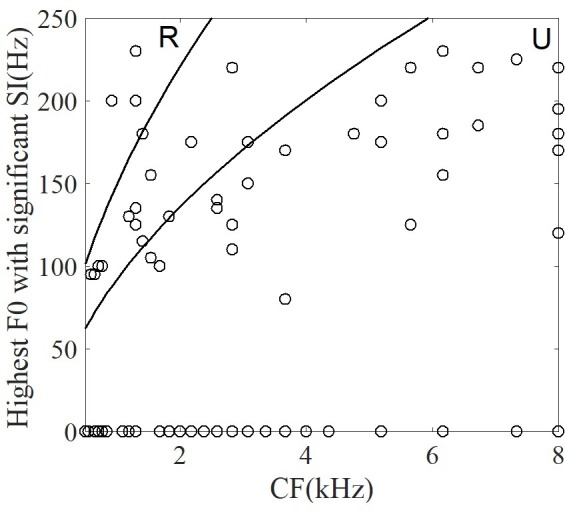
The correlation between the highest F0 with a significant SI and CF of single neuron. The two lines correspond to the range of resolvability (see ‘Discussion’). The harmonics of stimuli (upper left) around the CF would be resolved (‘R’ in figure), whereas the harmonics around CF (lower right) would be unresolved (‘U’ in figure).

For each neuron, the significantly synchronized highest F0 was defined as the maximum F0 among four tones and all the time segments. To examine whether the CF of single neurons was correlated with the significantly synchronized highest F0, the relationship between them are plotted in **Figure 10**. Some neurons (53% of neurons) were not synchronized at any stimulus F0. In **Figure 10** these neurons are plotted with highest F0 equal to 0. More than half of the remaining neurons (62% of 42 neurons) had significant synchronized highest F0 located as the range of unresolved harmonics (**Figure 10**, see ‘Discussion’), and the highest F0 was significantly positively correlated with the CF (*r* = 0.5, *p* < 0.001). However, the maximum SI at each neuron was not related to the CF (*r* = -0.17, *p* = 0.27). We suggest that periodicity of single IC neuron spike-timing patterns may be related to interactions of high-order frequency harmonics within the neuron’s pure tone response frequency area, such that the neuron may represent the F0 pitch associated with unresolved harmonics.

## Discussion

The aim of this study was to determine how the IC encodes time-variant F0 contours in Mandarin tones. The extent to which LFPs and single neuron activity represented the time-variant periodicity pitch of natural speech was examined in the IC of guinea pigs. In the time domain, LFPs reflected the time-variant fundamental period of speech, where there was consistency between interval bands and peak correlation bands in the autocorrelogram of response and stimulus. In the frequency domain, LFPs represented the F0 and harmonics, where the frequency bands with high spectral energy in the spectrogram of LFPs corresponded to the stimulus spectrogram. The F0 curves extracted from 93% of the responses significantly correlated with those extracted from the stimulus (*p* < 0.05), with 74% of the average linear pitch errors being lower than 10 Hz. These results suggested that LFPs recorded in IC code the F0 contours robustly.

For single neuron activity, phase-locking was also obvious both in the running all-order interspike interval distribution, where the most frequent interspike intervals corresponded to the time-variant fundamental period, and the Fourier transform of PSTHs, where the response significantly synchronized to F0 or 2F0 of the stimulus at corresponding time segments. While nearly all recorded LFPs could reflect F0 robustly, 47% (42/90) of single neurons significantly synchronized with the F0 or 2F0 of stimuli. These results are consistent with other studies suggesting LFPs reflect synchronized input activity (Denker et al., 2011), and that phase-locked temporal information is preserved in the IC (Gockel et al., 2011; Krishnan and Plack, 2011). Single neuron activity reflects the output of the IC processing of pitch information and, in our study we found that 47% of all recorded single neurons represented the F0 voice pitch robustly. To our knowledge, this is the first study to examine running all-order interval distributions of single IC neurons and to examine the correspondence between most frequent interspike intervals distribution and time-variant F0 contours in Mandarin speech.

### Phase-Locked LFPs of IC Represent the F0 Contours and Spectral Features

In our study, the latency of LFPs and the FRA of single neurons were used to check the recording position in the IC. The shape of LFPs responding to pure tones was consistent with a first positive peak at ∼6.5 ms across all the different frequencies (Orton et al., 2012), and the LFP morphology did not follow the stimulus waveform (as would be expected if the recording electrodes were directly picking up loudspeaker artifact). The latency (mean ± SD) of LFP responding to the speech stimuli was 6.6 ± 1.5 ms, and it was consistent with the previous studies (Harrison and Palmer, 1984). The FRA of single neuron responding to pure tones was similar with the study (Palmer et al., 2013).

In our study, we found that phase-locked LFPs recorded in the IC represented the time varying fundamental period in natural speech. When examining the autocorrelogram in the time domain, we found correspondence between the peak correlation bands in the autocorrelogram of LFPs and the stimuli (**Figure 3**). This result reinforces the predominant interval hypothesis for pitch encoding in the IC. The LFPs coding the F0 contours both in strength and direction (stimulus-to-response correlation) suggest that neural coding pitch in the IC are dynamic. Similar results have also been observed in scalp-recorded FFRs in Mandarin-speaking participants (Krishnan et al., 2004), suggesting that the temporal patterns of phase-locked activity of neural ensembles in the IC play a role in coding the F0 of complex speech.

In our study, we also found pitch strength of LFP responses depending on the tone type. The tone with a rising F0 produced significantly greater pitch strength than the flat tone and the falling tone. Although similar trends were also observed in other studies (Krishnan et al., 2004), the values of pitch strength found in the current study were relatively low compared to those reported in human studies (Mandarin-speaking participants; Krishnan et al., 2005). This result is also consistent with studies of neural responses to guinea pig vocalizations. Suta et al. (2003) reported that IC neurons’ firing rates were higher for whistle than time-inversed whistle, and suggested that the IC neurons of the guinea pig may prefer rising frequencies compared to falling frequencies.

The pitch strength was also significantly correlated with CF – the pitch strength increased as the CF increased. This result may be related to the relatively broader spectral tunning curve at the speech level used in this study. The bandwidth of tuning curves increase with the CF, and the harmonics and periodicity information coded by the LFP with high CF were therefore more robust.

### IC Single Neuron Activity Represent the F0 Voice Pitch Contours

In the current study, the most frequent intervals found in the running all-order intervals distribution of single IC neurons corresponded to the time-varying fundamental period of the stimuli. This finding was consistent with the studies of neural activity in the AN. Cariani and Delgutte (1996a,b) examined the AN fibers response to the time-variant F0 of synthesized vowel sounds. They were the first to pool interspike interval distributions of the population AN fibers and found the correspondence between most frequent interspike intervals and the human perceived pitch. They suggested that phase-locked neural activity is important for encoding the pitch of complex sounds. They also found that most single AN fibers could phase-lock to the pitch.

However, in the current study, we only found five single neurons in the IC with CFs higher than the F0 that clearly reflected the fundamental period information. This may be because the IC receives phase-locked information not only from CN (Frisina et al., 1990), but also from medial and lateral divisions of the superior olive (Oliver et al., 2003) and lateral lemniscus (Schreiner and Winer, 2005), and subsequently, the accuracy of phase-locking preserved in the AN and CN is reduced at the level of the IC.

One possible explanation for finding few IC neurons in this study is that temporal information may be transformed into a rate-place code in the IC (Cariani, 1999). This idea has been examined in studies of IC neurons’ responses to the periodicity of amplitude modulation signals (Langner and Schreiner, 1988). Langner and Schreiner (1988) tested the best modulation function of single IC neurons responding to different modulation frequencies with a carrier frequency at the CF of the neuron. They found that only ∼33% of the 225 single IC neurons could tune to a best modulation frequency when it is tested by synchronization, while almost 75% when it is tested by firing rate (Langner and Schreiner, 1988).

Another possible reason for finding few IC neurons that the most frequency interspike intervals in the running all-order intervals showing stimulus periodicity is we only used 10 presentations of each stimulus in our study, while Cariani and Delgutte (1996a) presented stimuli 100 times, thereby increasing their ability to visualize the pattern of most frequent intervals. Miller and colleagues collected at least 600 spikes (Miller and Sachs, 1983, 1984) while we only collected 200 spikes on average.

In the current study, the spectra of the PSTHs (**Figure 8**) of some neurons (47%) at specific time segments showed discharge patterns that were significantly synchronized to the F0, 2F0 or other harmonics. The best-synchronized frequency was generally below 500 Hz (**Figure 9**), and all synchronized frequencies were below the best modulation frequency limit of IC neurons responding to amplitude-modulated signals (Langner and Schreiner, 1988; Langner et al., 2002). Previous research has also suggested that a periodicity map might exist in IC (Schreiner and Langner, 1988; Langner et al., 2002; Schnupp et al., 2015), and our synchronized neurons may have been located across that map. Krishna and Semple (2000) found that the spike firing rate tuned to the modulation frequency in IC and the rate modulation function was not dependent on the synchronization modulation function (Langner and Schreiner, 1988; Müller-Preuss et al., 1994). These results suggest that both rate and synchronization representation of periodicity might exist at the level of the IC. Henry et al. (2017) also showed that IC neurons in awake budgerigar encoded the vowel-like sounds by the neural synchrony and variations in average discharge rate related the envelope structure. Studies of the correlation between the behaviourally measured sensitivity to amplitude-modulated signals and neural responses in the IC showed that the synchronized temporal representation was more correlated with behavioral performance than rate representation for envelope detection in awake budgerigar (Henry et al., 2016). Similar studies in awake rabbits, however, showed the opposite effect, with rate representation was more correlated to behavioral performance (Nelson and Carney, 2007).

The limit of upper modulation frequency synchronization, known to decrease progressively from auditory periphery to the central auditory system (Palmer, 1982; Schreiner and Urbas, 1988; Rees and Palmer, 1989; Joris and Yin, 1992, 1998; Rhode and Greenberg, 1994; Bieser and Müller-Preuss, 1996; Eggermont, 1998; Joris et al., 2004). Studies measuring from neurons in the auditory thalamus and auditory cortex have shown that the periodicity or repetition rate of temporally regular signals can be represented by the spiking timing (synchronized neurons) and/or average discharge-rate (non-synchronized neurons) (Lu et al., 2001; Lu and Wang, 2004; Bartlett and Wang, 2007). Bendor and Wang (2005) also found mixed-response neurons in the auditory cortex, and suggested that these neurons may play a role in temporal-to-rate transmission. The difference in the limits of neural synchronization between the IC (Batra et al., 1989), auditory thalamus (Bartlett and Wang, 2007) and auditory cortex (Lu et al., 2001) in awake animals suggests that the auditory thalamus may play the role of a transition stage for temporal-to-rate transformation (Wang et al., 2008), and that rate-place representation is more responsible than temporal representation for periodicity representation in auditory cortex (Bendor and Wang, 2005; Walker et al., 2011). It also has been suggested that the periodicity temporal code may transform to a rate-place code (Bartlett and Wang, 2007; Wang et al., 2008; Bendor and Wang, 2010; Plack et al., 2014; Langner, 2015) or spatial-temporal code (Loeb et al., 1983; Oxenham et al., 2004; Bernstein and Oxenham, 2005) in the upper auditory pathway. In the current study, 93% LFPs represented the time varying periodicity pitch, and only 47% single neurons represented the F0 pitch. The LFP reveal the synaptic input information, and single neurons reveal the axonal output information. The difference between the proportion of the LFPs and single neurons may suggest that the temporal coding transform to other form of neural coding.

The relationship between the CF of single neurons in guinea pig with the equivalent rectangular bandwidth (ERB) (ERB = 0.3 CF^0.56^, Shackleton et al., 2009) could be used to estimate whether any given stimulus contained resolved or unresolved components (**Figure 10**). In general, a sound can be said to contain only resolved harmonics when the ERB includes fewer than 2 harmonics, and unresolved when the ERB includes more than 3.25 harmonics (Shackleton and Carlyon, 1994). In the current study, the CF of most neurons corresponded to the unresolved harmonics. The results suggest that most neurons represent F0 voice pitch (**Figure 10**), and that this is due to the interaction of harmonics in the FRA of pure tones (Langner and Schreiner, 1988). In 47% of all recording neurons synchronized to the F0, 26 of 42 neurons (62%) had CFs located in the unresolved range. The unresolved range was decided by the stimulus F0. For a stimulus F0 of 100 Hz, 34% (26 of 76) of the recorded neurons were synchronized; and for a stimulus F0 of 150 Hz, 50% (23 of 46) were synchronized. The largest SI of each neuron was used to assess the ability of single neuron coding periodicity, and we found that there is no relationship between the CF of single neuron and the largest SI (*r* = -0.17, *p* = 0.27). The results suggest that there is no relationship between the proportion of synchronized neurons in the resolved and unresolved ranges in current study, and the results was consistent with the study (Shackleton et al., 2009). One possible reason is that the sound level was 80 dB SPL, and the FRA at this level may have included more stimulus harmonics even though the CF of the neuron were located in the range between the resolved and unresolved. Although the amplitude of the stimuli was changing with the time, the largest SI of all synchronized neurons at each time segment was not correlated with the root mean square of the stimulus amplitude (*r* = -0.28, *p* = 0.17).

Three of the neurons we recorded did have CFs corresponding to resolved harmonics. The maximum SI of these three neurons in the resolved harmonics was not significant different in the unresolved harmonics. Other researchers have found similar results in studies of IC neurons in response to harmonic complex tones (Shackleton et al., 2009). However, there are some discrepancies between our results and others. Sinex and Li (2007) examined IC neurons’ responses to single harmonic tones with an F0 of 250 Hz and found that the discharge pattern of single neurons was modulated at F0, but only in some cases. Shackleton et al. (2009) tested multi-unit activity from the IC with different F0s (50, 100, 200, 400 Hz) of harmonic tones and found that most clusters were synchronized to F0 above 280 Hz. Compared with their studies, differences include the stimuli and the response analysis time windows used. In the study by Shackleton et al. (2009), synthesized harmonic tones with fixed F0 were used. In our study, complex speech sounds with time-variant F0 from 80 to 220 Hz were used (**Figure 1C**). Another difference is the method used in the two studies: Shackleton et al. (2009) recorded multiunit responses to stimuli of 100 ms duration, and the entire response waveform was analyzed. In Sinex and Li (2007), the stimulus was 500 ms and the response after 100 ms was analyzed. They found that the discharge pattern was generally not related to the F0, except in some cases in which the discharge pattern was modulated by an F0 of 250 Hz. However, in our study the stimulus duration ranged from of 400 to 600 ms, and the response was analyzed in 50 ms successive time segments. As a result, the difference of synchronized frequency components may be due to the time windows of analysis.

### Method Limitations

Although the results of LFPs are similar to FFRs in human studies, an important limitation is that the Mandarin tone is not a natural or behaviourally relevant sound to the guinea pig. As previously discussed, spike rate is affected by a sound’s relevance to behavior (Suta et al., 2003). Urethane is a common anesthetic for acute recordings of neural responses in the auditory midbrain; however, studies have demonstrated that it inhibits spontaneous and evoked spike rate (Albrecht and Davidowa, 1989; Capsius and Leppelsack, 1996). Other studies suggested that there is no significant effect on spectral tuning properties in the anesthetic state (Schumacher et al., 2011; Palmer et al., 2013). The phase-locking to the F0 was recorded in the IC of urethane anesthetic guinea pig in our study, so as the study by Shackleton et al. (2009). The CF of most neurons was higher than 1 kHz and corresponded to the unresolved harmonics of stimulus, so our results only showed that single neurons represent the F0 pitch of stimulus.

## Author Contributions

FP, WH, and YZ designed the experiments. FP, XW, and NH performed the experiments and collected the data. FP, HI-B, CM, and JF analyzed the data and interpreted results of experiments. FP, HI-B, and JF drafted the manuscript. All authors edited and revised manuscript critically and approved final version of manuscript.

## Conflict of Interest Statement

The authors declare that the research was conducted in the absence of any commercial or financial relationships that could be construed as a potential conflict of interest.

## References

[B1] Albrecht D. Davidowa H. (1989). Action of urethane on dorsal lateral geniculate neurons. *Brain Res. Bull.* 22 923–927. 10.1016/0361-9230(89)90001-4 2790498

[B2] Andoni S. Li N. Pollak G. D. (2007). Spectrotemporal receptive fields in the inferior colliculus revealing selectivity for spectral motion in conspecific vocalizations. *J. Neurosci.* 27 4882–4893. 10.1523/JNEUROSCI.4342-06.2007 17475796 PMC6672083

[B3] Bartlett E. L. Wang X. (2007). Neural representations of temporally modulated signals in the auditory thalamus of awake primates. *J. Neurophysiol.* 97 1005–1017. 10.1152/jn.00593.2006 17050830

[B4] Batra R. Kuwada S. Stanford T. R. (1989). Temporal coding of envelopes and their interaural delays in the inferior colliculus of the unanesthetized rabbit. *J. Neurophysiol.* 61 257–268. 10.1152/jn.1989.61.2.257 2918354

[B5] Bendor D. Wang X. (2005). The neuronal representation of pitch in primate auditory cortex. *Nature* 436 1161–1165. 10.1038/nature03867 16121182 PMC1780171

[B6] Bendor D. Wang X. (2007). Differential neural coding of acoustic flutter within primate auditory cortex. *Nat. Neurosci.* 10 763–771. 10.1038/nn1888 17468752

[B7] Bendor D. Wang X. (2008). Neural response properties of primary, rostral, and rostrotemporal core fields in the auditory cortex of marmoset monkeys. *J. Neurophysiol.* 100 888–906. 10.1152/jn.00884.2007 18525020 PMC2525707

[B8] Bendor D. Wang X. (2010). Neural coding of periodicity in marmoset auditory cortex. *J. Neurophysiol.* 103 1809–1822. 10.1152/jn.00281.2009 20147419 PMC2853289

[B9] Bernstein J. G. Oxenham A. J. (2005). An autocorrelation model with place dependence to account for the effect of harmonic number on fundamental frequency discrimination. *J. Acoust. Soc. Am.* 117 3816–3831. 10.1121/1.1904268 16018484 PMC1451417

[B10] Bidelman G. M. Gandour J. T. Krishnan A. (2011). Cross-domain effects of music and language experience on the representation of pitch in the human auditory brainstem. *J. Cogn. Neurosci.* 23 425–434. 10.1162/jocn.2009.21362 19925180

[B11] Bieser A. Müller-Preuss P. (1996). Auditory responsive cortex in the squirrel monkey: neural responses to amplitude-modulated sounds. *Exp. Brain Res.* 108 273–284. 10.1007/BF00228100 8815035

[B12] Bones O. Hopkins K. Krishnan A. Plack C. J. (2014). Phase locked neural activity in the human brainstem predicts preference for musical consonance. *Neuropsychologia* 58 23–32. 10.1016/j.neuropsychologia.2014.03.011 24690415 PMC4040538

[B13] Brimijoin W. O. O’Neill W. E. (2005). On the prediction of sweep rate and directional selectivity for FM sounds from two-tone interactions in the inferior colliculus. *Hear. Res.* 210 63–79. 10.1016/j.heares.2005.07.005 16263230 PMC3901414

[B14] Capsius B. Leppelsack H. J. (1996). Influence of urethane anesthesia on neural processing in the auditory cortex analogue of a songbird. *Hear. Res.* 96 59–70. 10.1016/0378-5955(96)00038-X 8817307

[B15] Cariani P. (1999). Temporal coding of periodicity pitch in the auditory system: an overview. *Neural Plast.* 6 147–172. 10.1155/NP.1999.147 10714267 PMC2565322

[B16] Cariani P. A. Delgutte B. (1996a). Neural correlates of the pitch of complex tones. I. pitch and pitch salience. *J. Neurophysiol.* 76 1698–1716. 10.1152/jn.1996.76.3.1698 8890286

[B17] Cariani P. A. Delgutte B. (1996b). Neural correlates of the pitch of complex tones. II. pitch shift, pitch ambiguity, phase invariance, pitch circularity, rate pitch, and the dominance region for pitch. *J. Neurophysiol.* 76 1717–1734. 10.1152/jn.1996.76.3.1717 8890287

[B18] Chandrasekaran B. Kraus N. (2010). The scalp-recorded brainstem response to speech: neural origins and plasticity. *Psychophysiology* 47 236–246. 10.1111/j.1469-8986.2009.00928.x 19824950 PMC3088516

[B19] Chandrasekaran B. Kraus N. Wong P. C. (2012). Human inferior colliculus activity relates to individual differences in spoken language learning. *J. Neurophysiol.* 107 1325–1336. 10.1152/jn.00923.2011 22131377 PMC3311681

[B20] Cunningham J. Nicol T. King C. Zecker S. G. Kraus N. (2002). Effects of noise and cue enhancement on neural responses to speech in auditory midbrain, thalamus and cortex. *Hear. Res.* 169 97–111. 10.1016/S0378-5955(02)00344-1 12121743

[B21] De Boer E. (ed.). (1976). “On the “residue” and auditory pitch perception,” in *Auditory System*, Berlin: Springer 479–583.

[B22] Denker M. Roux S. Linden H. Diesmann M. Riehle A. Grun S. (2011). The local field potential reflects surplus spike synchrony. *Cereb. Cortex* 21 2681–2695. 10.1093/cercor/bhr040 21508303 PMC3209854

[B23] Eggermont J. J. (1998). Representation of spectral and temporal sound features in three cortical fields of the cat. Similarities outweigh differences. *J. Neurophysiol.* 80 2743–2764. 10.1152/jn.1998.80.5.2743 9819278

[B24] Evans E. (1978). Place and time coding of frequency in the peripheral auditory system: some physiological pros and cons. *Audiology* 17 369–420. 10.3109/00206097809072605 697652

[B25] Fallon J. B. Irving S. Pannu S. S. Tooker A. C. Wise A. K. Shepherd R. K. (2016). Second spatial derivative analysis of cortical surface potentials recorded in cat primary auditory cortex using thin film surface arrays: comparisons with multi-unit data. *J. Neurosci. Methods* 267 14–20. 10.1016/j.jneumeth.2016.04.004 27060384 PMC4884480

[B26] Frisina R. D. Smith R. L. Chamberlain S. C. (1990). Encoding of amplitude modulation in the gerbil cochlear nucleus: I. A hierarchy of enhancement. *Hear. Res.* 44 99–122. 10.1016/0378-5955(90)90074-Y 2329098

[B27] Gockel H. E. Carlyon R. P. Mehta A. Plack C. J. (2011). The frequency following response (FFR) may reflect pitch-bearing information but is not a direct representation of pitch. *J. Assoc. Res. Otolaryngol.* 12 767–782. 10.1007/s10162-011-0284-1 21826534 PMC3214239

[B28] Goldberg J. M. Brownell W. E. (1973). Discharge charateristics of neurons in anteroventral and dorsal cochlear nuclei of cat. *Brain Res.* 64 35–54. 10.1016/0006-8993(73)90169-8 4360881

[B29] Greenberg S. (1981). *Neural Temporal Coding of Pitch and Vowel Quality: Human Frequency-Following Response Studies of Complex Signals.* Los Angeles, CA: Phonetics Laboratory Department of Linguistics.

[B30] Harrison R. Palmer A. (1984). Neurone response latency in the inferior colliculus in relation to the auditory brainstem responses (ABR) in the guinea pig. *Scand. Audiol.* 13 275–281. 10.3109/01050398409042136 6523046

[B31] Henry K. S. Abrams K. S. Forst J. Mender M. J. Neilans E. G. Idrobo F. (2017). Midbrain synchrony to envelope structure supports behavioral sensitivity to single-formant vowel-like sounds in noise. *J. Assoc. Res. Otolaryngol.* 18 165–181. 10.1007/s10162-016-0594-4 27766433 PMC5243265

[B32] Henry K. S. Neilans E. G. Abrams K. S. Idrobo F. Carney L. H. (2016). Neural correlates of behavioral amplitude modulation sensitivity in the budgerigar midbrain. *J. Neurophysiol.* 115 1905–1916. 10.1152/jn.01003.2015 26843608 PMC4869485

[B33] Jeng F. C. Hu J. Dickman B. Montgomery-Reagan K. Tong M. Wu G. (2011). Cross-linguistic comparison of frequency-following responses to voice pitch in American and Chinese neonates and adults. *Ear Hear.* 32 699–707. 10.1097/AUD.0b013e31821cc0df 21543983

[B34] Joris P. Schreiner C. Rees A. (2004). Neural processing of amplitude-modulated sounds. *Physiol. Rev.* 84 541–577. 10.1152/physrev.00029.2003 15044682

[B35] Joris P. X. Yin T. C. (1992). Responses to amplitude-modulated tones in the auditory nerve of the cat. *J. Acoust. Soc. Am.* 91 215–232. 10.1121/1.402757 1737873

[B36] Joris P. X. Yin T. C. (1998). Envelope coding in the lateral superior olive. III. Comparison with afferent pathways. *J. Neurophysiol.* 79 253–269. 10.1152/jn.1998.79.1.253 9425196

[B37] King C. Nicol T. Mcgee T. Kraus N. (1999). Thalamic asymmetry is related to acoustic signal complexity. *Neurosci. Lett.* 267 89–92. 10.1016/S0304-3940(99)00336-5 10400219

[B38] Kraus N. Mcgee T. Carrell T. King C. Littman T. Nicol T. (1994). Discrimination of speech-like contrasts in the auditory thalamus and cortex. *J. Acoust. Soc. Am.* 96 2758–2768. 10.1121/1.411282 7983281

[B39] Krishna B. S. Semple M. N. (2000). Auditory temporal processing: responses to sinusoidally amplitude-modulated tones in the inferior colliculus. *J. Neurophysiol.* 84 255–273. 10.1152/jn.2000.84.1.255 10899201

[B40] Krishnan A. Gandour J. T. Bidelman G. M. (2010). The effects of tone language experience on pitch processing in the brainstem. *J. Neurolinguistics* 23 81–95. 10.1016/j.jneuroling.2009.09.001 20161561 PMC2805250

[B41] Krishnan A. Plack C. J. (2011). Neural encoding in the human brainstem relevant to the pitch of complex tones. *Hear. Res.* 275 110–119. 10.1016/j.heares.2010.12.008 21167923

[B42] Krishnan A. Xu Y. Gandour J. Cariani P. (2005). Encoding of pitch in the human brainstem is sensitive to language experience. *Brain Res. Cogn. Brain Res.* 25 161–168. 10.1016/j.cogbrainres.2005.05.004 15935624

[B43] Krishnan A. Xu Y. Gandour J. T. Cariani P. A. (2004). Human frequency-following response: representation of pitch contours in Chinese tones. *Hear. Res.* 189 1–12. 10.1016/S0378-5955(03)00402-7 14987747

[B44] Langner G. Albert M. Briede T. (2002). Temporal and spatial coding of periodicity information in the inferior colliculus of awake chinchilla (Chinchilla laniger). *Hear. Res.* 168 110–130. 10.1016/S0378-5955(02)00367-2 12117514

[B45] Langner G. Schreiner C. E. (1988). Periodicity coding in the inferior colliculus of the cat. I. Neuronal mechanisms. *J. Neurophysiol.* 60 1799–1822. 10.1152/jn.1988.60.6.1799 3236052

[B46] Langner G. D. (2015). *The Neural Code of Pitch and Harmony.* Cambridge: University Press 10.1017/CBO9781139050852

[B47] Licklider J. C. R. (1951). A duplex theory of pitch perception. *J. Acoust. Soc. Am.* 23 147–147. 10.1121/1.1917296 14831572

[B48] Liu L. F. Palmer A. R. Wallace M. N. (2006). Phase-locked responses to pure tones in the inferior colliculus. *J. Neurophysiol.* 95 1926–1935. 10.1152/jn.00497.2005 16339005

[B49] Loeb G. E. White M. W. Merzenich M. M. (1983). Spatial cross-correlation. *Biol. Cybern.* 47 149–163. 10.1007/BF00337005 6615914

[B50] Lu T. Liang L. Wang X. (2001). Temporal and rate representations of time-varying signals in the auditory cortex of awake primates. *Nat. Neurosci.* 4 1131–1138. 10.1038/nn737 11593234

[B51] Lu T. Wang X. (2004). Information content of auditory cortical responses to time-varying acoustic stimuli. *J. Neurophysiol.* 91 301–313. 10.1152/jn.00022.2003 14523081

[B52] Malone B. J. Scott B. H. Semple M. N. (2007). Dynamic amplitude coding in the auditory cortex of awake rhesus macaques. *J. Neurophysiol.* 98 1451–1474. 10.1152/jn.01203.2006 17615123

[B53] Mandarin L. S. (2015). *The Mandarin Words Tested in the Mandarin Level Test.* Available at: http://www.pthxx.com/e_download/02_zici_1/index.html

[B54] Mardia K. V. (1975). Statistics of directional data. *J. R. Stat. Soc. Series B* 37 349–393.

[B55] Miller M. I. Sachs M. B. (1983). Representation of stop consonants in the discharge patterns of auditory-nerve fibers. *J. Acoust. Soc. Am.* 74 502–517. 10.1121/1.389816 6619427

[B56] Miller M. I. Sachs M. B. (1984). Representation of voice pitch in discharge patterns of auditory-nerve fibers. *Hear. Res.* 14 257–279. 10.1016/0378-5955(84)90054-6 6480513

[B57] Moore B. C. (2012). *An Introduction to the Psychology of Hearing.* Netherlands: Brill.

[B58] Müller-Preuss P. Flachskamm C. Bieser A. (1994). Neural encoding of amplitude modulation within the auditory midbrain of squirrel monkeys. *Hear. Res.* 80 197–208. 10.1016/0378-5955(94)90111-2 7896578

[B59] Nakamoto K. T. Shackleton T. M. Palmer A. R. (2010). Responses in the inferior colliculus of the guinea pig to concurrent harmonic series and the effect of inactivation of descending controls. *J. Neurophysiol.* 103 2050–2061. 10.1152/jn.00451.2009 20147418 PMC2853265

[B60] Nelson P. C. Carney L. H. (2007). Neural rate and timing cues for detection and discrimination of amplitude-modulated tones in the awake rabbit inferior colliculus. *J. Neurophysiol.* 97 522–539. 10.1152/jn.00776.2006 17079342 PMC2577033

[B61] Nordmark J. O. (1978). Frequency and periodicity analysis. *Handb. Percept.* 4 243–282. 10.1016/B978-0-12-161904-6.50014-2

[B62] Oliver D. L. Beckius G. E. Bishop D. C. Loftus W. C. Batra R. (2003). Topography of interaural temporal disparity coding in projections of medial superior olive to inferior colliculus. *J. Neurosci.* 23 7438–7449. 10.1523/JNEUROSCI.23-19-07438.2003 12917380 PMC6740450

[B63] Orton L. D. Poon P. W. Rees A. (2012). Deactivation of the inferior colliculus by cooling demonstrates intercollicular modulation of neuronal activity. *Front. Neural Circuits* 6:100. 10.3389/fncir.2012.00100 23248587 PMC3522070

[B64] Oxenham A. J. Bernstein J. G. Penagos H. (2004). Correct tonotopic representation is necessary for complex pitch perception. *Proc. Natl. Acad. Sci. U.S.A.* 101 1421–1425. 10.1073/pnas.0306958101 14718671 PMC337068

[B65] Palmer A. R. (1982). Encoding of rapid amplitude fluctuations by cochlear-nerve fibres in the guinea-pig. *Eur. Arch. Otorhinolaryngol.* 236 197–202. 10.1007/BF00454039 7150083

[B66] Palmer A. R. Russell I. J. (1986). Phase-locking in the cochlear nerve of the guinea-pig and its relation to the receptor potential of inner hair-cells. *Hear. Res.* 24 1–15. 10.1016/0378-5955(86)90002-X 3759671

[B67] Palmer A. R. Shackleton T. M. Sumner C. J. Zobay O. Rees A. (2013). Classification of frequency response areas in the inferior colliculus reveals continua not discrete classes. *J. Physiol.* 591 4003–4025. 10.1113/jphysiol.2013.255943 23753527 PMC3764642

[B68] Peng F. Xia N. Wang X. Zheng X. L. Zhou Y. Fan X.Y. (2016). Neural representation of different mandarin tones in the inferior colliculus of the guinea pig. *Conf. Proc. IEEE Eng. Med. Biol. Soc.* 2016 1608–1611. 10.1109/EMBC.2016.7591020 28268636

[B69] Perez C. A. Engineer C. T. Jakkamsetti V. Carraway R. S. Perry M. S. Kilgard M. P. (2013). Different timescales for the neural coding of consonant and vowel sounds. *Cereb. Cortex* 23 670–683. 10.1093/cercor/bhs045 22426334 PMC3563339

[B70] Pettersen K. H. Lindén H. Dale A. M. Einevoll G. T. (2012). Extracellular spikes and CSD. *Handb. Neural Act. Meas.* 1 92–135. 10.1017/CBO9780511979958.004

[B71] Plack C. J. Barker D. Hall D. A. (2014). Pitch coding and pitch processing in the human brain. *Hear. Res.* 307 53–64. 10.1016/j.heares.2013.07.020 23938209

[B72] Plomp R. (1967). Pitch of complex tones. *J. Acoust. Soc. Am.* 41 1526–1533. 10.1121/1.1910515 6075560

[B73] Ranasinghe K. G. Vrana W. A. Matney C. J. Kilgard M. P. (2013). Increasing diversity of neural responses to speech sounds across the central auditory pathway. *Neuroscience* 252 80–97. 10.1016/j.neuroscience.2013.08.005 23954862 PMC3795858

[B74] Rees A. Palmer A. R. (1989). Neuronal responses to amplitude-modulated and pure-tone stimuli in the guinea pig inferior colliculus, and their modification by broadband noise. *J. Acoust. Soc. Am.* 85 1978–1994. 10.1121/1.397851 2732379

[B75] Rhode W. S. (1995). Interspike intervals as a correlate of periodicity pitch in cat cochlear nucleus. *J. Acoust. Soc. Am.* 97 2414–2429. 10.1121/1.411963 7714259

[B76] Rhode W. S. Greenberg S. (1994). Encoding of amplitude modulation in the cochlear nucleus of the cat. *J. Neurophysiol.* 71 1797–1825. 10.1152/jn.1994.71.5.1797 8064349

[B77] Sayles M. Winter I. M. (2008). Reverberation challenges the temporal representation of the pitch of complex sounds. *Neuron* 58 789–801. 10.1016/j.neuron.2008.03.029 18549789

[B78] Schnupp J. W. Garcia-Lazaro J. A. Lesica N. A. (2015). Periodotopy in the gerbil inferior colliculus: local clustering rather than a gradient map. *Front. Neural Circuits* 9:37. 10.3389/fncir.2015.00037 26379508 PMC4550179

[B79] Schreiner C. Winer J. A. (2005). *The Inferior Colliculus.* Berlin: Springer.

[B80] Schreiner C. E. Langner G. (1988). Periodicity coding in the inferior colliculus of the cat. II. Topographical organization. *J. Neurophysiol.* 60 1823–1840. 10.1152/jn.1988.60.6.1823 3236053

[B81] Schreiner C. E. Urbas J. V. (1988). Representation of amplitude modulation in the auditory cortex of the cat. II. Comparison between cortical fields. *Hear. Res.* 32 49–63. 10.1016/0378-5955(88)90146-3 3350774

[B82] Schumacher J. W. Schneider D. M. Woolley S. M. (2011). Anesthetic state modulates excitability but not spectral tuning or neural discrimination in single auditory midbrain neurons. *J. Neurophysiol.* 106 500–514. 10.1152/jn.01072.2010 21543752 PMC3154814

[B83] Shackleton T. M. Carlyon R. P. (1994). The role of resolved and unresolved harmonics in pitch perception and frequency modulation discrimination. *J. Acoust. Soc. Am.* 95 3529–3540. 10.1121/1.409970 8046144

[B84] Shackleton T. M. Liu L. F. Palmer A. R. (2009). Responses to diotic, dichotic, and alternating phase harmonic stimuli in the inferior colliculus of guinea pigs. *J. Assoc. Res. Otolaryngol.* 10 76–90. 10.1007/s10162-008-0149-4 19089495 PMC2644390

[B85] Sinex D. G. (2008). Responses of cochlear nucleus neurons to harmonic and mistuned complex tones. *Hear. Res.* 238 39–48. 10.1016/j.heares.2007.11.001 18078726 PMC2323903

[B86] Sinex D. G. Li H. (2007). Responses of inferior colliculus neurons to double harmonic tones. *J. Neurophysiol.* 98 3171–3184. 10.1152/jn.00516.2007 17913991 PMC2649952

[B87] Small A. M. (1970). “Periodicity pitch,” in *Foundations of Modern Auditory Theory*, ed. Tobias J. V. (New York, NY: Academic Press).

[B88] Smith J. C. Marsh J. T. Brown W. S. (1975). Far-field recorded frequency-following responses: evidence for the locus of brainstem sources. *Electroencephalogr. Clin. Neurophysiol.* 39 465–472. 10.1016/0013-4694(75)90047-4 52439

[B89] Snyder R. L. Bierer J. A. Middlebrooks J. C. (2004). Topographic spread of inferior colliculus activation in response to acoustic and intracochlear electric stimulation. *J. Assoc. Res. Otolaryngol.* 5 305–322. 10.1007/s10162-004-4026-5 15492888 PMC2504547

[B90] Song J. H. Skoe E. Wong P. C. Kraus N. (2008). Plasticity in the adult human auditory brainstem following short-term linguistic training. *J. Cogn. Neurosci.* 20 1892–1902. 10.1162/jocn.2008.20131 18370594 PMC2829864

[B91] Steadman M. (2015). *Investigating the Neural Code for Dynamic Speech and the Effect of Signal Degradation.* Nottingham: University of Nottingham.

[B92] Suta D. Kvasnak E. Popelar J. Syka J. (2003). Representation of species-specific vocalizations in the inferior colliculus of the guinea pig. *J. Neurophysiol.* 90 3794–3808. 10.1152/jn.01175.2002 12944528

[B93] Syka J. Aitkin L. (1981). *Neuronal Mechanisms of Hearing.* New York, NY: Plenum 10.1007/978-1-4684-3908-3

[B94] Tao C. Zhang G. Zhou C. Wang L. Yan S. Zhang L. I. (2016). Synaptic basis for the generation of response variation in auditory cortex. *Sci. Rep.* 6:31024. 10.1038/srep31024 27484928 PMC4971572

[B95] van Noorden L. (1982). “Two channel pitch perception,” in *Music, Mind, and Brain*, ed. Clynes M. (Berlin: Springer), 251–269. 10.1007/978-1-4684-8917-0_13

[B96] Walker K. M. Bizley J. K. King A. J. Schnupp J. W. (2011). Cortical encoding of pitch: recent results and open questions. *Hear. Res.* 271 74–87. 10.1016/j.heares.2010.04.015 20457240 PMC3098378

[B97] Wallace M. N. Anderson L. A. Palmer A. R. (2007). Phase-locked responses to pure tones in the auditory thalamus. *J. Neurophysiol.* 98 1941–1952. 10.1152/jn.00697.2007 17699690

[B98] Wallace M. N. Shackleton T. M. Palmer A. R. (2002). Phase-locked responses to pure tones in the primary auditory cortex. *Hear. Res.* 172 160–171. 10.1016/S0378-5955(02)00580-4 12361879

[B99] Wang X. Lu T. Bendor D. Bartlett E. (2008). Neural coding of temporal information in auditory thalamus and cortex. *Neuroscience* 157 484–493. 10.1016/j.neuroscience.2008.07.050 19143093

[B100] Wang X. Lu T. Liang L. (2003). Cortical processing of temporal modulations. *Speech Commun.* 41 107–121. 10.1016/S0167-6393(02)00097-3

[B101] Warren R. M. (2013). *Auditory Perception: A New Synthesis.* New York, NY: Elsevier.

[B102] White-Schwoch T. Nicol T. Warrier C. M. Abrams D. A. Kraus N. (2016). Individual differences in human auditory processing: insights from single-trial auditory midbrain activity in an animal model. *Cereb. Cortex* 27 5095–5115. 10.1093/cercor/bhw293 28334187 PMC6410521

[B103] Williams A. J. Fuzessery Z. M. (2010). Facilitatory mechanisms shape selectivity for the rate and direction of FM sweeps in the inferior colliculus of the pallid bat. *J. Neurophysiol.* 104 1456–1471. 10.1152/jn.00598.2009 20631213 PMC2944671

[B104] Winter I. M. Palmer A. R. (1990). Responses of single units in the anteroventral cochlear nucleus of the guinea pig. *Hear. Res.* 44 161–178. 10.1016/0378-5955(90)90078-4 2329092

[B105] Wong P. C. Skoe E. Russo N. M. Dees T. Kraus N. (2007). Musical experience shapes human brainstem encoding of linguistic pitch patterns. *Nat. Neurosci.* 10 420–422. 10.1038/nn1872 17351633 PMC4508274

[B106] Woolley S. M. Casseday J. H. (2005). Processing of modulated sounds in the zebra finch auditory midbrain: responses to noise, frequency sweeps, and sinusoidal amplitude modulations. *J. Neurophysiol.* 94 1143–1157. 10.1152/jn.01064.2004 15817647

[B107] Worden F. G. Marsh J. T. (1968). Frequency-following (microphonic-like) neural responses evoked by sound. *Electroencephalogr. Clin. Neurophysiol.* 25 42–52. 10.1016/0013-4694(68)90085-0 4174782

